# One Sea, Different Whales: Genomics Sheds Light on a Small Population of Fin Whales

**DOI:** 10.1093/gbe/evag084

**Published:** 2026-04-28

**Authors:** Roberto Biello, Alessio Iannucci, Silvia Fuselli, Elisa Desiato, Jorge Urban R., Maria Cristina Fossi, Cristina Panti, Annalaura Mancia

**Affiliations:** Department of Life Science and Biotechnology, University of Ferrara, Ferrara 44121, Italy; Department of Biology, University of Florence, Sesto Fiorentino 50019, Italy; Department of Life Science and Biotechnology, University of Ferrara, Ferrara 44121, Italy; Department of Life Science and Biotechnology, University of Ferrara, Ferrara 44121, Italy; Department of Biology, University of Florence, Sesto Fiorentino 50019, Italy; Departamento de Ciencias Marinas y Costeras, Universidad Autónoma de Baja California Sur, La Paz 23081, México; Department of Physical Sciences, Earth and Environment, University of Siena, Siena 53100, Italy; Department of Physical Sciences, Earth and Environment, University of Siena, Siena 53100, Italy; Department of Life Science and Biotechnology, University of Ferrara, Ferrara 44121, Italy; Department of Biology and Marine Science, Marine Science Research Institute, Jacksonville University, Jacksonville, FL 32211, USA; Department of Biology, University of North Florida, Jacksonville, FL 32224, USA

**Keywords:** *Balaenoptera physalus*, conservation, genetic load, genetic diversity, cetacean, inbreeding, population genomics, genome assembly and annotation

## Abstract

Whales are key components of marine ecosystems, and several populations are affected by environmental and anthropogenic pressures. Among them, the fin whale, *Balaenoptera physalus*, from the Mediterranean Sea remains poorly characterized at the genomic level despite its classification as Endangered and ongoing population decline driven by human-induced habitat degradation. While bioacoustics and telemetry studies suggest the presence of both resident and migratory subgroups, the extent of genetic isolation in this population remains unclear. Here, we present the first whole-genome analysis of Mediterranean fin whales to assess genomic variability, genetic load, population structure, and adaptive potential of an immunity locus. By comparing genomes from the Mediterranean with those of fin whales from the North Atlantic, North Pacific, and new sequences from Sea of Cortez, we evaluated the degree of genetic isolation and placed the Mediterranean population within a broader evolutionary and conservation context. Our results show that although Mediterranean fin whales form a distinct genetic cluster, they are not fully isolated from North Atlantic populations. We also detected genetic heterogeneity within the Mediterranean basin, with some individuals showing no admixture and others displaying a substantial ancestry component from a different cluster, consistent with previous observations of subgroups exhibiting different migratory tendencies. Despite showing moderate genomic diversity and some adaptive potential compared to other populations, the Mediterranean population remains vulnerable to genomic erosion due to demographic decline, limited connectivity, and growing environmental stress. These findings underscore the importance of conservation actions and long-term genomic monitoring.

Significance Despite its ecological importance and Endangered status, the genomic characteristics and population structure of the Mediterranean fin whale (*Balaenoptera physalus*) remain largely unknown. By sequencing and comparing whole genomes from Mediterranean and global populations, we reveal low but persistent gene flow with North Atlantic whales and uncover fine-scale substructure within the Mediterranean basin. Although this small population exhibits levels of diversity, inbreeding, genetic load, and adaptive immune gene variation comparable to North Atlantic populations, signs of demographic decline and isolation suggest that continued conservation attention and long-term genetic monitoring may be warranted.

## Introduction

Whales have long captured human fascination, not only due to their massive size but also because of their complex behaviors, migratory patterns, and ecological importance. Among them, baleen whales play a crucial role in maintaining ocean ecosystems by facilitating nutrient cycling and influencing marine food webs (Durfort et al. 2022; Ganley et al. 2022). However, many species face increasing environmental pressures, from climate change to human-induced disturbances, making their study more critical than ever (Panti et al. 2019; Tulloch et al. 2019; Fossi et al. 2020; Peters et al. 2022).

As the climate crisis accelerates, genomic studies have become important tools for investigating population structure, evolutionary history, and patterns of isolation. By analyzing genetic differentiation, we can determine whether a population is truly isolated or maintains some connectivity with neighboring groups. This approach is essential for conservation efforts, as isolated populations with low genetic diversity may be more vulnerable to environmental changes, disease, pollution, and habitat degradation (Simmonds and Eliott 2009; Cabrera et al. 2021). Furthermore, understanding the degree of isolation can clarify the evolutionary processes shaping populations, providing insights into how adaptation occurs across different ecological contexts (Clapham et al. 1999; Pallin et al. 2023).

The fin whale (*Balaenoptera physalus*) is the second-largest whale species and mainly inhabits temperate and polar latitudes, with an apparent equatorial hiatus separating Northern and Southern Hemisphere populations (Edwards et al. 2015; Aguilar and García-Vernet 2018). Like most mysticetes, fin whales typically undertake long-range annual migrations with a seasonal cycle between high-latitude feeding grounds and low-latitude breeding and calving grounds in winter, where feeding is absent (Kellogg 1929; Katona and Whitehead 1981). As an exception to these migration patterns, resident populations of fin whales, such as those in the Sea of Cortez and the Mediterranean Sea, have also been documented (Tershy et al. 1993; Bérubé et al. 1998; Forney and Barlow 1998; Bérubé et al. 2002; Palsbøll et al. 2004; Urbán et al. 2005; Moore et al. 2006; Stafford et al. 2007; Mizroch et al. 2009).

The Mediterranean fin whale population has been identified as distinct from the North Atlantic populations through bioacoustics and telemetry studies (Bentaleb et al. 2011; Cotté et al. 2011; Castellote et al. 2012; Panigada et al. 2017). Moreover, previous studies suggested the presence of both resident and migratory subgroups within the Mediterranean Sea, adding complexity to its structure (Castellote et al. 2012). To date, the genetic characterization of this fin whale population remains poor, with only a few studies based on traditional molecular markers conducted to determine whether this population is genetically isolated (Bérubé et al. 1998; Palsbøll et al. 2004). These studies suggest that Mediterranean fin whales are genetically differentiated from North Atlantic whales, with patterns more consistent with recurrent gene flow than with complete isolation.

Fin whales in the Mediterranean Sea are known to aggregate during the summer months in the Pelagos Sanctuary for Marine Mammals, a marine protected area in the northwestern Mediterranean that is key feeding ground and an important habitat for several cetacean species regularly occurring in the region (Notabartolo di Sciara et al. 2003; Coll et al. 2012; Ham et al. 2021). During winter, these whales are thought to migrate toward the southern part of the basin (Panigada et al. 2011). Despite its high biodiversity and the presence of protected areas, the Mediterranean Sea is subject to intense anthropogenic pressures, including climate change, habitat degradation, heavy maritime traffic, noise pollution, and contamination from chemical pollutants such as microplastics (Collignon et al. 2012; Fossi et al. 2013, 2016; Cózar et al. 2015; Espada et al. 2024). These threats, combined with their restricted distribution within the semi-enclosed and heavily impacted Mediterranean basin and an inferred continuing decline in the number of mature individuals, recently estimated at around 2,500, have led to their classification as Endangered on the IUCN Red List of Threatened Species (Panigada et al. 2021).

Given the limited genomic knowledge and the threats faced by fin whales, this study provides the first genomic characterization of the Mediterranean fin whale population, aiming to more accurately reconstruct its population structure, as well as its demographic and evolutionary history. In particular, we inferred the potential for adaptation and vulnerability to increasing environmental disturbances by using the estimated level of genomic variability within the Mediterranean population and genetic load as proxies. Furthermore, comparison with previously published data (nine samples from the North Pacific, five from the Sea of Cortez, 11 from Iceland, and seven from Svalbard) (Wolf et al. 2022; Nigenda-Morales et al. 2023), together with new data generated in this study from populations of the same species in different geographical areas (13 samples from the Mediterranean Sea and an additional seven from the Sea of Cortez), provided a dataset comprising 52 individuals in total. This allowed us to: (i) assess the degree of isolation of the Mediterranean population from North Atlantic populations, with which some level of gene flow is hypothesized, and (ii) contextualize Mediterranean fin whale genomes within a broad geographical framework. This framework includes North Pacific populations, known for their relatively large population sizes (Nigenda-Morales et al. 2023), as well as North Atlantic fin whales from Iceland, whose severe demographic declines driven by historical exploitation provide a reference for populations affected by human impact (Wolf et al. 2022). Additionally, the Sea of Cortez population was included, as it provides an important point of comparison with a nearly completely isolated and small-sized population (Nigenda-Morales et al. 2023).

## Results

### The Fin Whale Reference Genome

**Fig. 1.**
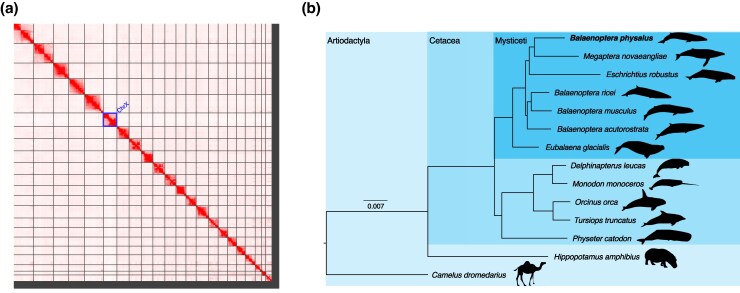
The fin whale reference genome. a) HiC heat-map of genomic interactions. Interactions between two locations are depicted by a red pixel. Black lines depict scaffold boundaries for the 22 chromosome-length scaffolds; the X chromosome is labeled ChrX. b) Maximum likelihood phylogeny of the fin whale and 11 other Cetacea species. The tree is rooted with *Hippopotamus amphibius* and *Camelus dromedarius*. Branch lengths are in amino acid substitutions per site.

The previous versions of the fin whale (*Balaenoptera physalus*) genome assemblies (Yim et al. 2014; Wolf et al. 2022; https://www.dnazoo.org/assemblies/balaenoptera_physalus) had a total length between 2.4 and 2.7 Gb (Table 1) but differ in their suitability for downstream analyses. To obtain a chromosome-level reference with improved anchoring, we scaffolded the Wolf et al. (2022) assembly using publicly available Hi-C data generated from a female individual (https://www.dnazoo.org/assemblies/balaenoptera_physalus; SRR16970344). This produced a final assembly of 12,892 scaffolds with an N50 of 109.1 Mb and an L50 of 9 (Table 1). After manual curation, the final assembly comprised 2.410 Gb, with 96.9% of the assembly anchored into 22 chromosomes (21 autosomes + X chromosome) (Fig. 1a, Fig. S1, Table 1), consistent with the fin whale 2n karyotype being 42 chromosomes + XY chromosomes (Árnason 1969). Based on coverage analyses, we discovered the sex chromosome in the genome assembly (Fig. S2), showing 50% lower coverage than the autosomes. The lengths of the 22 chromosomes ranged from 182 to 35 Mb. The final assembly has a BUSCO completeness score of 93.5% using the Mammalia gene set, a per-base quality (QV) of 29.05 and a k-mer completeness of 91.62. Thus, the new assembly (Bphy_ph2.v2) represents a near-complete and highly contiguous assembly and a significant improvement on the existing assemblies of this species (Table 1). The identification of repetitive elements resulted in a 38.2% repeat content, falling within the range of repeat contents for other Cetacean species (Fan et al. 2019; Tollis et al. 2019). In total, 25,321 protein-coding genes were predicted. The BUSCO completeness of the gene annotation using the same Mammalia gene set was 89.8%. To place the genome assembly in a phylogenetic context, we compared the proteome of *B. physalus* (which includes the complete set of annotated protein-coding genes) to those of 12 other cetacean species with fully sequenced genomes (see Table S1). Using a maximum likelihood approach, we conducted a phylogenetic analysis based on 5,464 conserved single-copy genes (Fig. 1b). The tree confirmed the closer phylogenetic relationship of *B. physalus* with the humpback whale (*Megaptera novaeangliae*) compared to other *Balaenoptera* species, consistent with previous phylogenetic analyses (Wolf et al. 2023).

**Table 1:** Comparison of assembly metrics between the fin whale genome generated in this study and publicly available fin whale genome assemblies

Genome Assembly	*Baphy*	*SBiKF_Bphy_ph2*	*Balaenoptera_physalus_HiC*	*Bphy_ph2.v2*
**Reference**	Yim et al. (2014)	Wolf et al. (2022)	DNA Zoo	This study
**GenBank/ENA accession #**	GCA_008795845.1	GCA_023338255.1	/	/
**Base pairs (Gb)**	2.462	2.41	2.754	2.41
**Total scaffolds**	62,302	13,140	1,361,899	12,892
**Scaffold N50 (Mb)**	0.9	24.9	77.5	109.1
**Scaffold L50**	803	27	13	9
**Longest scaffold (Mb)**	6.8	91.4	156.9	182.7
**Assembly in chromosomes**	0.00%	0.00%	72.70%	96.90%

### Population Structure

Population structure was initially investigated using mitogenomes to enable direct comparison with the abundant data already available in the literature (20 individuals sequenced in this study and an additional 265 retrieved from published sources). The phylogenetic analysis of complete mitogenomic haplotypes revealed three main clades that are strongly supported by bootstrap values and associated with distinct ocean basins (Fig. S3). Cluster 1 primarily included haplotypes from the North Pacific (NPA) and the Sea of Cortez (SOC). Cluster 2 consisted of haplotypes from the Southern Hemisphere, along with two smaller clusters from the North Atlantic and NPA. Cluster 3 included samples from the North Atlantic and the Mediterranean Sea (Fig. S3). These results are consistent with those obtained in previous studies (Archer et al. 2013; Cabrera et al. 2019; Buss et al. 2023).

**Fig. 2.**
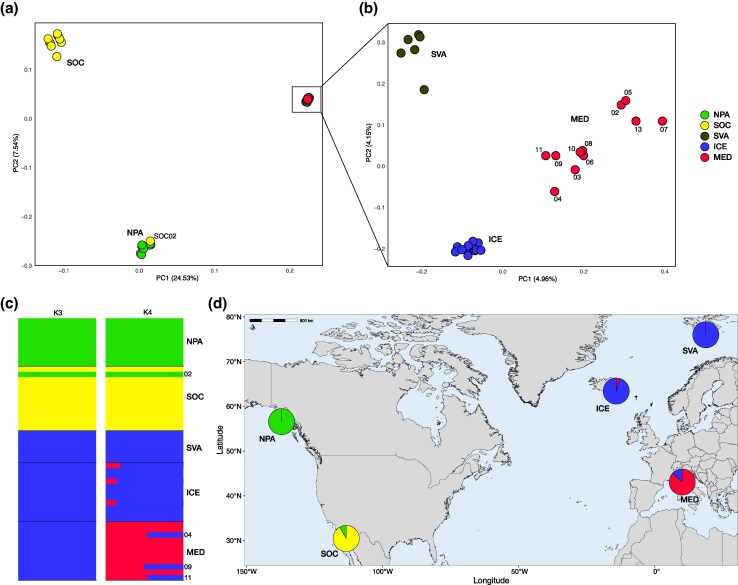
Population structure. PCA on the entire dataset a) and excluding individuals from the Sea of Cortez (SOC) and the North Pacific (NPA) b). c) Individual-level ADMIXTURE results for *K* = 3 and *K* = 4; each bar represents an individual, and colors indicate the proportion of ancestry assigned to each cluster. d) Geographic distribution of the five macro-areas from which samples were collected (circles); pie charts show ADMIXTURE-inferred ancestry proportions for K = 4 (see panel labels).

In addition to the mitochondrial analyses, we examined nuclear genome variation using whole-genome data from 49 individuals (Tables S2 and S3). We excluded three sibling individuals, retaining only sibling pairs from SOC, because close kinship appeared common in this population (see *Relatedness* in *Materials and Methods* and Table S4). Principal component analysis (PCA) revealed distinct patterns of genetic differentiation across populations. Along the first principal component (PC1; 24.53% of the variance explained), Pacific and Atlantic populations separated clearly, underscoring substantial divergence between these two oceanic regions. Further examination within the Pacific populations revealed additional structure along PC2 (7.54% of the variance explained), where the isolated population from the SOC was clearly distinct from the NPA samples (Fig. 2a). Importantly, one SOC individual clustered with NPA (Fig. 2a), a pattern also observed in Nigenda-Morales et al. (2023), consistent with occasional movement between regions. We then performed a second PCA restricted to the Atlantic and Mediterranean samples. This analysis separated the Iceland (ICE), Svalbard (SVA), and Mediterranean (MED) groups (PC1 = 4.96% and PC2 = 4.15% of the variance explained; Fig. 2b). Within these populations, the MED samples exhibited the highest level of internal genetic variation (Figs. S4–S6). The admixture analysis agreed with these findings, revealing a similar genetic structure (Fig. 2c–d). At K = 2, the most strongly supported number of genetic clusters (Fig. S7), there was a clear distinction between Atlantic and Pacific populations, highlighting the deep genetic divergence between these oceanic regions (Fig. S8). However, NPA also showed evidence of shared ancestry with the Atlantic populations (ICE and SVA; Fig. S8). Increasing the number of clusters to K = 4 identified four distinct genetic groups: one corresponding to the MED group, another to the SOC, a third including the NPA population, and a fourth cluster that included both the ICE and SVA populations (Fig. 2c–d). At K = 5, the ICE and SVA populations remained distinct clusters from MED, although a few MED samples still showed shared ancestry with them (Fig. S8). Interestingly, the three admixed MED samples (MED04, MED09, MED11) are those located closest to the ICE cluster in the PCA (Fig. 2b–c). Consistent with the PCA, the SOC individual clustering with NPA was also supported by the admixture results.

Pairwise F_ST_ analysis revealed a relatively high level of genetic differentiation between the SOC population and all other populations (above 0.15) (Table S5). High genetic differentiation was also observed between the NPA population and the North Atlantic and Mediterranean populations (above 0.17). In contrast, F_ST_ values among the MED, ICE, and SVA populations were consistently low, all falling below 0.03. To assess the extent of internal genetic differentiation of the Mediterranean group in terms of F_ST_ relative to the other populations, MED individuals were divided into admixed (MED_adm) and non-admixed (MED_noadm) groups. The results indicate that the MED_noadm group represents the most genetically differentiated group within the Mediterranean–North Atlantic framework (Table S5).

### Genomic Diversity and Inbreeding

To investigate genome-wide diversity and inbreeding across fin whale populations, we estimated *θ_w_*, examined runs of homozygosity (ROH), and calculated the fraction of the genome in ROH (FROH).

**Fig. 3.**
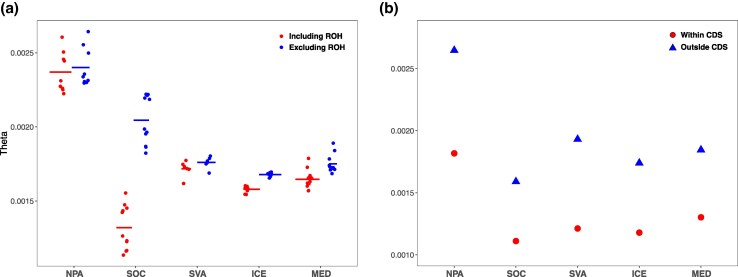
Genomic diversity (Watterson's Theta, *θ_w_*). a) Individual-level *θ_w_* estimated with ROHan, reported including ROH and outside ROH. b) Population-level *θ_w_* per site estimated with ANGSD for putatively neutral regions (outside CDS) and exonic regions (within CDS).

In particular, *θ_w_* values ranged from the highest in NPA to the lowest in SOC (Fig. 3a). Since ROH are genomic regions characterized by low variability, their inclusion in diversity estimates is expected to reduce genome-wide values. However, the extent of this reduction may differ across populations, reflecting variation in demographic history and levels of inbreeding. The inclusion of ROH regions led to a reduction in diversity estimates of 1.25% in NPA, 2.46% in SVA, 5.91% in ICE, 6.01% in MED, and a marked 35.58% in SOC, consistent with expectations for a small population subject to recent inbreeding (Fig. 3a). Genomic diversity estimates in both neutral regions (outside CDS) and CDS showed a lower diversity in SOC and higher in NPA population (Fig. 3b). In contrast, populations in the Atlantic Ocean and Mediterranean Sea showed similar values. The degree of divergence of the *θ_w_* estimate between neutral regions and CDS was higher in SVA (59.52%) and lower in MED (41.91%) (Fig. 3b).

**Fig. 4.**
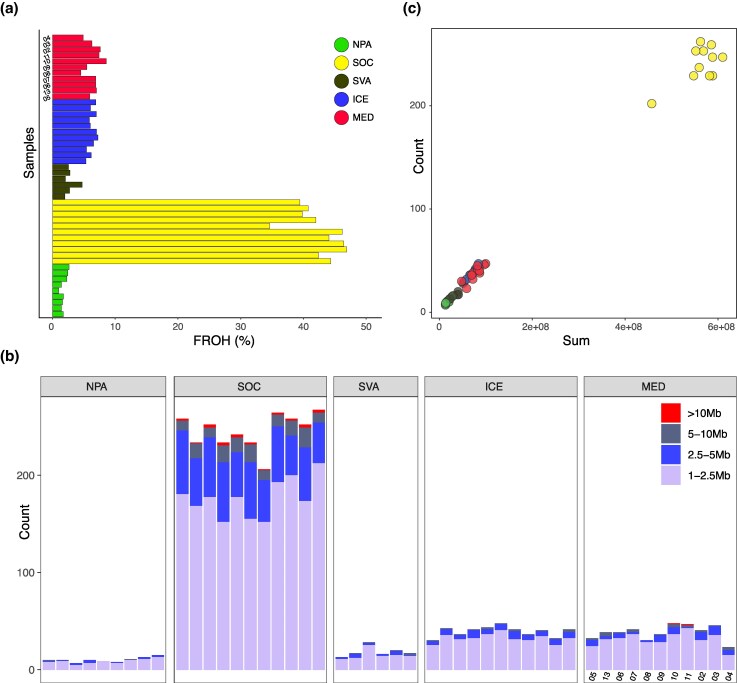
Inbreeding in fin whale populations. a) The fraction of the genome in ROH (>1 Mb) estimated with ROHan. Color is representative of the ROH size in megabases (Mb). b) Number of ROH compared to the sum of the length of ROH across the autosomes.

The fraction of the genome being in ROH (FROH) was consistent within each population. The SOC population had the highest mean FROH (39.17%). Lower FROH levels were observed in the populations of MED, ICE and SVA, with a mean FROH value of 6.50%, 6.32% and 2.80%, respectively. The lowest level of FROH was seen in NPA, which has a mean FROH value of 1.81% (Fig. 4a). In comparison to the other populations, the SOC population showed a high number of ROH larger than 2.5 Mb (Fig. 4b). ICE and MED populations also showed several ROH above 2.5 Mb, slightly higher than SVA and NPA (Fig. 4b).

To investigate recent demographic history, we analyzed the sum and number of runs of homozygosity (SROH and NROH, respectively) following the approach described in Foote et al. (2021). In our samples, SROH and NROH within individual genomes were highly correlated (Spearman correlation *r* = 0.879, *P* = 2.2e-16). Notably, the SOC population showed particularly high levels of both metrics, consistent with a history of inbreeding and a reduced effective population size. Within the MED and the ICE populations, some individuals also showed relatively higher values. Interestingly, the other North Atlantic population, SVA, shows a pattern similar to that of the wider NPA (Fig. 4c, Table S6).

### MHC *DQB-1* Genetic Variation

We additionally analyzed exon 2 of the MHC *DQB-1* locus, the most variable region, as an indicator of a genomic region in which greater intra- and inter-individual diversity reflects enhanced adaptative potential and immune response to pathogens (Bernatchez and Landry 2003; Nigenda-Morales et al. 2008). MHC DQB-1 exon 2 was analyzed in fin whale genomes from the different populations included in this work (NPA, SOC, SVA, ICE, and MED), together with orthologous sequences from public repositories for two closely related species (*Balaenoptera musculus*, MUS, and *Megaptera novaeangliae*, MEG) (Table S7). Given the challenges in accurately annotating the highly complex MHC region, we chose this locus because we were able to annotate it manually. Basic indices of genetic diversity suggested that all analyzed fin whale populations showed substantial levels of variation at a key locus involved in pathogen interaction. In particular, in this genomic region, *θ_w_*, a measure of the population mutation rate, was ten times higher than in the rest of the coding regions in the case of NPA, and even twenty times higher in all other cases (Fig. 3).

### Demographic History

**Fig. 5.**
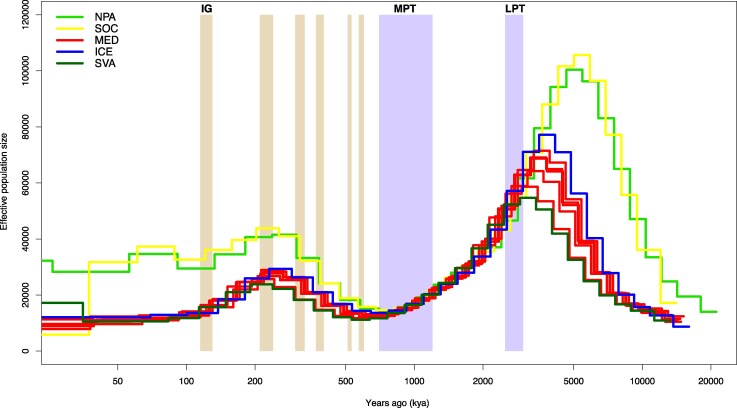
MSMC2 effective population size estimates. The x axis shows the time, and the y axis shows Ne. The model covers the last 20 Ma to 30 kya and is scaled based on a mutation rate of 1.38 × 10−8 per site per generation (Árnason et al. 2018) and a generation time of 25.9 years. The mid-Pleistocene transition (MPT, 0.7 to 1.2 Ma) and the Late-Pleistocene Transition (LPT, 2.6 Ma) are shown as the light blue shaded region, light red shading indicates interglacials (IG) in the Pleistocene and Holocene.

To test whether demographic histories of fin whale populations reflect major climatic transitions, we inferred changes in effective population size (Ne) over the past 20 million years using the multiple sequentially Markovian coalescent (MSMC2) approach (Schiffels and Wang 2020). We hypothesized that fin whale populations from different ocean basins could have followed similar demographic trajectories, given that glacial–interglacial cycles represent global climatic events. However, ancestral effective population sizes for Pacific populations (NPA and SOC) were notably higher before the Plio-Pleistocene transition (LPT, 2.6 million years ago, Ma) compared to both recent estimates and those inferred for North Atlantic (ICE and SVA) and Mediterranean populations (Fig. 5 and S9). After the mid-Pleistocene transition (MPT), Ne for all populations slightly increased until approximately 100 to 200 thousand years ago (ka), which coincides with the last interglacial periods (Fig. 5). Following this time, fin whale populations in the Atlantic Ocean (ICE and SVA) and MED declined, whereas populations from NPA and SOC remained stable (Fig. 5). More recently, the SOC population has experienced a decline (Fig. 5).

### Genetic Load

We investigated patterns of genetic load in 40 samples with at least 10× coverage (Tables S2 and S3), using *B. musculus* and *M. novaeangliae* as outgroups to polarize SNPs.

**Fig. 6.**
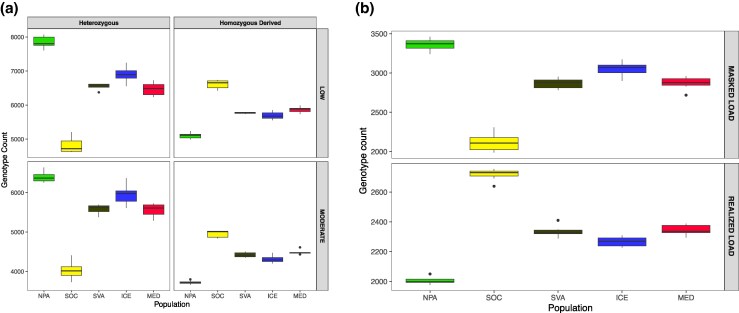
Genetic load. a) Proportion of heterozygous genotypes and homozygous derived genotypes for synonymous sites (LOW) and missense sites (MODERATE) in different fin whale populations. b) Genetic load partitioned into masked load (top; deleterious derived alleles in heterozygous genotypes, GERP > 4) and realized load (bottom; deleterious derived alleles in homozygous genotypes, GERP > 4).

We first calculated the mutational load based on functional annotations from SNP effects predicted by snpEff (Cingolani et al. 2012). This analysis allowed us to assess potentially deleterious variation in two impact categories: moderate impact (missense) and low impact (synonymous). Both categories exhibited similar patterns of homozygous and heterozygous derived genotypes across the five populations (Fig. 6a), with heterozygosity reduced and homozygosity elevated in the SOC population.

We further assessed potentially deleterious effects using conservation-based GERP scores, which provide quantitative estimates of deleteriousness. When examining variants in coding regions, which are more likely to have functional consequences, we observed a similar trend based on GERP scores (Fig. 6b). MED, SVA, and ICE showed comparable levels of genetic load, whereas NPA differs mainly in the balance between masked and realized components (Fig. 6b). Specifically, MED exhibits a lower masked load, but a higher realized load compared to NPA (Fig. 6b). The higher realized load in SOC, reflecting an increase in homozygous deleterious alleles, suggests that a larger fraction of deleterious variation is unmasked by homozygosity and therefore more likely to contribute to fitness effects in this population. This may be attributed to their smaller population size, where genetic drift likely plays a significant role (Bertorelle et al. 2022).

## Discussion

The Mediterranean fin whale population is classified as Endangered. It has long been considered genetically distinct from the North Atlantic populations, although the degree of isolation and the boundaries between these populations remain subjects of debate.

Using a whole-genome approach, we found that Mediterranean fin whales are not completely genetically isolated. While the Mediterranean population forms a distinct genetic cluster separated from the North Atlantic populations, our results point to a more complex internal structure. In particular, ongoing gene flow with North Atlantic populations appears to involve only a subset of individuals, whereas others seem more genetically isolated. This pattern is consistent with occasional or seasonal movements through the Strait of Gibraltar, as previously proposed (Gauffier et al. 2009, 2018; Bentaleb et al. 2011; Castellote et al. 2012). It also supports the hypothesis that some fin whales observed in the Mediterranean Sea may represent a summer feeding group that migrates elsewhere for winter breeding. Our genetic results align with the evidence from bioacoustics studies, which identified two contrasting song patterns of fin whales in the Mediterranean Sea. These findings have been interpreted as reflecting two different populations with contrasting migratory strategies, one seasonally connected to the Atlantic and the other resident year-round (Castellote et al. 2012; Sciacca et al. 2015; Notabartolo di Sciara et al. 2016; Pereira et al. 2020). Taken together, these lines of evidence suggest that the more genetically distinct individuals identified here may correspond to a resident Mediterranean subpopulation (Fig. 2). Future work combining higher-coverage genomes with explicit demographic modelling will be essential to formally quantify the timing and magnitude of divergence and ongoing gene flow between Mediterranean and North Atlantic populations, and to test alternative scenarios underlying the hypothesized within-Mediterranean substructure.

The MED population showed levels of heterozygosity and inbreeding comparable to those of the North Atlantic populations. This indicates that, although there may be some genetic isolation, the MED population is not facing extreme signs of genomic erosion, unlike the more isolated SOC population (Figs. 3 and 4). One possible explanation is that ongoing or recent gene flow has been sufficient to maintain relatively stable genetic diversity, even though the Mediterranean and North Atlantic populations remain genetically distinguishable. However, given the long generation times of the species and its recent decline (Panigada et al. 2021), we cannot exclude the possibility that the genomic consequences of recent demographic events are not yet fully apparent. A similar explanation was proposed by Wolf et al. (2022) for the weak bottleneck signal observed in some metrics in the ICE population, including relatively preserved heterozygosity, despite the documented severe genetic bottleneck caused by whaling, which peaked over the last two centuries (Aguilar and García-Vernet 2018; Wolf et al. 2022).

To investigate how such changes might manifest at functionally important genes, we focused on the MHC DQB-1 locus. Considering the exceptionally high level of polymorphism concentrated in the exon 2 of most MHC loci, this genomic region represents a reasonable compromise between the reliable identification of genetic variation and the detection of meaningful evolutionary signals. Despite the inherent limitations of analyzing a single locus, our results suggest that all the analyzed populations retain a relatively high genetic capacity for pathogen resistance, an important factor for long-term viability (Table S7). The recent detection of Cetacean Morbillivirus (CeMV) infections in MED fin whales highlights the importance of avoiding the erosion of genetic diversity at MHC loci that may occur during demographic decline (Sutton et al. 2011, 2015; Zhang et al. 2016; Vargas-Castro et al. 2023), especially considering that this virus also induces immunosuppression through MHC downregulation (Mazzariol et al. 2016; Gaur et al. 2024).

In this context, we investigated demographic histories and found slightly different trajectories between Pacific and Atlantic fin whale populations (Fig. 5). Pacific populations (NPA and SOC) maintained larger ancestral effective population sizes (Ne), particularly during the Plio-Pleistocene transition (∼2.6 Ma). In contrast, Atlantic (ICE and SVA) and Mediterranean (MED) populations exhibited lower ancestral Ne and experienced a smaller recovery following the mid-Pleistocene transition (∼700 to 200 ka). These different trends likely reflect differences in connectivity and environmental conditions across these ocean basins. Because MSMC2 infers changes in the inverse coalescence rate, apparent Ne changes can also be influenced by population structure and temporal variation in migration rates, not only by changes in population size (e.g. Mazet et al. 2016). Although MSMC2 is not accurate for recent times (Hilgers et al. 2025), the inferred long-term decline in Ne for North Atlantic and MED populations is consistent with historically reduced effective sizes and/or changes in connectivity over time. These patterns agree with previous genomic studies (Yim et al. 2014; Árnason et al. 2018; Wolf et al. 2022; Nigenda-Morales et al. 2023).

The MED fin whales exhibited similar levels of masked and realized load with North Atlantic populations but differed from NPA and SOC populations (Fig. 6). The MED population exhibited a lower masked load compared to NPA, but a higher realized load. This pattern has already been observed in the SOC and NPA populations of fin whales and is consistent with reduced genome-wide heterozygosity and small population sizes (Nigenda-Morales et al. 2023). What we report here for the first time is that populations from the North Atlantic (ICE and SVA) and MED showed similar levels among themselves and an intermediate genetic load between SOC (small population) and NPA (large population). The higher realized load in SOC, reflecting an increase in homozygous deleterious alleles, suggested that these mutations are fully expressed in this population. These results imply that SOC individuals have genomes carrying a higher realized load compared to the MED population. In the Mediterranean basin, multiple anthropogenic threats are persistent and often difficult to mitigate. Under these conditions, future consanguineous mating could unmask more deleterious mutations in MED, and its relatively low effective population size and restricted connectivity may increase risk if environmental or demographic pressures intensify.

Despite some ongoing gene flow with the North Atlantic and a comparable level of genomic diversity, inbreeding, genetic load and adaptive variation, the Mediterranean fin whale population faces substantial risks due to its small size, partial isolation, and vulnerability to environmental changes. Rising temperatures and anthropogenic stressors may disrupt migration, feeding, and reproduction, leading to a lower input of novel genetic variants, affecting both potential evolutionary change and genetic load (Silber et al. 2017; Bertorelle et al. 2022; Kebke et al. 2022; Nigenda-Morales et al. 2023). Moreover, suppose resident individuals will be forced to alter their geographic range, as observed in other fin whale populations (Ruiz-Sagalés et al. 2024). In this context, behavioral plasticity, including shifts in diet and migratory behavior, may allow Mediterranean fin whales to exploit new habitats, potentially altering the structure and functioning of the ecosystem they currently inhabit (Pearson et al. 2023).

Conservation efforts should prioritize protecting Mediterranean fin whale habitats and reducing human-induced threats. Future studies should continue to monitor genetic diversity and migration patterns by expanding the area of sampling, as well as assessing how environmental changes impact the delicate balance between Mediterranean and North Atlantic fin whale populations.

## Materials and Methods

### Genome Scaffolding and Annotation

Because the two previously available *B. physalus* genome assemblies each had complementary limitations, the assembly from Wolf et al. (2022) was high quality but not chromosome-scale, whereas the DNA ZOO assembly (https://www.dnazoo.org/assemblies/balaenoptera_physalus) was chromosome-scale but contained a substantial amount of unplaced sequence. We scaffolded the assembly from Wolf et al. (2022) with DNA ZOO data to obtain a chromosome-level assembly better suited for downstream analyses, particularly the identification and treatment of sex-linked regions. These data include in vivo Hi-C data (SRR16970344). Chromap v0.2.4 (Zhang et al. 2021) was used to align the HiC reads to the genome using parameters for HiC data (−preset hic) and remove PCR duplicates (−remove-pcr-duplicates). The output file was converted to a sorted BAM file with samtools v1.11 (Li et al. 2009). The latter, along with the reference assembly, served as the input files for scaffolding with YaHS v1.2 (Zhou et al. 2023). This was implemented using standard options, and YaHS outputs were converted using the “juicer_pre” function of YaHS to Juicebox Assembly Tools (jbat) (Dudchenko et al. 2018) compatible files for manual curation visually within Juicebox. Following manual curation, edits were applied to the scaffold assembly using “juicer_post”. The quality of the genome assembly was assessed by searching for conserved, single-copy, Mammalia genes (*n* = 9,226) with Benchmarking Universal Single-Copy Orthologs (BUSCO) v5.3.2 (Simão et al. 2015) and by analysis of k-mer spectra with MERQURY (Rhie et al. 2020) to compare k-mer content of the raw sequencing reads to the assembly k-mer content.

Using the homology-based analysis, we identified the known transposable elements (TE) within the *B. physalus* genome using Repeatmasker v4.0.7 (Smit et al. 2013) with the combined database of RepBase (Jurka et al. 2005) and Dfam Consensus (Wheeler et al. 2012). Repeatmasker was launched with options “-e ncbi -species mammalia -xsmall -gff” (Jurka et al. 2005).

For gene prediction, RNA-seq reads available on NCBI from various tissues of closely related species were downloaded (Table S8). Quality control and trimming for adapters and low-quality bases (quality score <20) of the raw reads were performed using fastqc v0.11.8 (Andrews 2010) and TrimGalore v0.5.0 (https://github.com/FelixKrueger/TrimGalore), respectively. High-quality reads were then mapped to the soft-masked assembly with HISAT2 v2.2.1 (Kim et al. 2015) and sorted with samtools v1.11 (Li et al. 2009). All the BAM files were filtered to remove invalid splice junctions with Portcullis v1.2.4 (Mapleson et al. 2018). Filtered RNA-seq alignments were passed to Braker v3 (Quinlan 2014; Kovaka et al. 2019; Pertea and Pertea 2020; Bruna et al. 2024; Gabriel et al. 2024), together with protein sequences of nine closely related species from the order Artiodactyla, including two *Balaenoptera* species (Table S9). The Braker gene prediction pipeline was run with the options “–softmasking”. This pipeline uses StringTie (Pertea et al. 2015) to assemble the RNA-seq reads, followed by rounds of GeneMark and AUGUSTUS training and gene prediction (Stanke and Waack 2003). Gene sets were combined with TSEBRA (Gabriel et al. 2021). The completeness of the final gene set was checked with BUSCO v5.3.2 (Simão et al. 2015) using the longest transcript of each gene as the representative transcript.

Sequences were searched against the nonredundant NCBI protein database using DIAMOND v0.9.10 (Buchfink et al. 2015) with an E-value cut-off of ≤ 1 × 10^−5^. BLAST2GO v5.0 (Conesa et al. 2005) and INTERPROSCAN v2.5.0 (Quevillon et al. 2005) were used to assign Gene Ontology (GO) terms. The protein domains were annotated by searching against the InterPro v32.0 (Hunter et al. 2012) and Pfam v27.0 (Punta et al. 2012) databases, using INTERPROSCAN v5.52 (Quevillon et al. 2005) and HMMER v3.3 (Finn et al. 2014), respectively.

### Genetic Sexing from Coverage

Reads from one male (MED11) and one female (MED08) were aligned to the reference genome with BWA-MEM v0.7.17 (Li 2013) using default settings. Alignments were sorted and indexed in SAMtools v1.11 (Li et al. 2009), and per-base depth was obtained with samtools depth. Mean coverage was summarized per scaffold for each sex, and male:female coverage ratios were computed to identify sex-linked scaffolds. Plots were generated in R (R Core Team) to visualize scaffold-level coverage patterns.

### Phylogeny

Orthologous groups in Cetacea genomes were identified from the predicted protein sequences of *B. physalus* and 11 other Cetacea genomes already published (see Table S1). As outgroups, the genomes of two Artyodactyla were included: *Camelus dromedarius* (GCF_036321535.1) and *Hippopotamus amphibius kiboko* (GCF_030028045.1) (Table S1). The longest transcript was used to represent the gene model when several transcripts of a gene were annotated. Orthofinder v2.5.4 (Emms and Kelly 2019) together with diamond v0.9.14 (Buchfink et al. 2015), Multiple Alignment using Fast Fourier Transform (MAFFT) v7.305 (Katoh and Standley 2013) and RAxML v8.2.12 (Stamatakis 2014), run with default parameters, was used to cluster proteins into orthogroups, reconstruct gene trees and estimate the species tree.

### Sampling, DNA Isolation and Sequencing

A total of 13 tissue samples were collected as skin biopsies from individual free-ranging fin whales in the Mediterranean Sea (MED) in 2018 to 2019 (Table S2). Samples were collected by remote dart-sampling, by national and international regulations, and under sampling permits n. 0018799/PNM released to the University of Siena from the Italian Ministry of Environment and Energy Safety and the Italian National Institute for Environmental Protection and Research (ISPRA). The research permits included the necessary ethical approval for sample collection, analysis, and use. Additional individuals sampled in the Sea of Cortez were sequenced in this study (SOC, *N* = 7; Table S2). Skin tissues obtained from darts were snap-frozen in liquid nitrogen and then stored at −80 °C until processing. DNA was extracted from 20 mg of skin tissue using the Wizard® Genomic DNA Purification Kit (Promega) following the manufacturer's instructions. DNA integrity was assessed by electrophoresis on a 1% agarose gel, and DNA concentration was measured by fluorometric analysis using the Qubit™ 4 Fluorometer (Invitrogen, Waltham, USA). Short-read genomic libraries were constructed using an Illumina DNA PCR-Free Prep Kit (Illumina) according to the manufacturer's protocol. For sequencing, libraries were run paired-end 2 × 150 bp using an Illumina NovaSeq 6000 System (Illumina, Inc., San Diego, CA, USA) on a S1 flow cell with 500 Gb as final output.

### Mapping

Demultiplexing and conversion of sequencing data from bcl to fastq formats were performed using bcl2fastq v2.20 (Illumina). Quality control of the reads was performed with FastQC v0.11.8 (Andrews 2010). Reads were then processed with AdapterRemoval v2 (Schubert et al. 2016) to remove residual Illumina adapters. Filtered reads were aligned to the *B. physalus* reference genome produced in this study using the mem algorithm implemented in the bwa v0.7.15 aligner (Li and Durbin 2009). Alignments in sam format were sorted, indexed and compressed in bam format using samtools v1.9 (Li and Durbin 2009). PCR duplicates were removed, produced during library preparation, and optical duplicates using the MarkDuplicates tool in the Picard Toolkit v2.18.20 (http://broadinstitute.github.io/picard/). Regions close to indels showing putative alignment errors were identified and realigned using the RealignerTargetCreator and the IndelRealignment tools in GATK v3.5 (McKenna et al. 2010). Alignment statistics were calculated using the CollectAlignmentSummaryMetrics tool, and bam files were validated with the ValidateSamFile tool of the Picard Toolkit v2.18.20. Observed coverage was computed using the depth command of samtools v1.9 with the “-aa” flag activated.

Additionally, paired-end reads of 27 additional samples (see Table S3) of *B. physalus* sampled from Iceland (ICE, *N* = 11), Svalbard archipelago (SVA, *N* = 7), North Pacific (NPA, *N* = 9) and 5 additional SOC individuals were downloaded and processed using the same bioinformatic pipeline described above. The ICE samples from Wolf et al. (2022) were subsampled to reduce potential bias due to strongly unbalanced population sample sizes in downstream analyses and were restricted to individuals collected in 2019 to improve temporal comparability with the Mediterranean dataset.

### Mitochondrial DNA

Raw reads of the 20 individuals sequenced in this work were aligned to the fin whale mitogenome reference sequence (Árnason et al. 1991; NCBI access no. NC001321) as described above. Consensus sequences were created with angsd v0.932 using options -minQ 20 -minMapQ 30 -setMinDepth 3. Additional complete mitogenome sequences were retrieved from the literature (Table S10) for a total of 265 sequences. Sequences were aligned using Geneious Prime 2020.1.1 (Kearse et al. 2012), visually checked for accuracy and trimmed to the shortest available sequence (16,423 bp).

All 265 sequences were used to build an mtDNA phylogenetic tree using RAxML v8.2.7 implemented in Geneious Prime 2020.1.1 by applying the GTR GAMMA nucleotide model with rapid bootstrapping and search for best-scoring maximum-likelihood trees across 100 bootstrap replicates (Stamatakis 2014). The complete mtDNA sequence of the *Balaenoptera musculus* outgroup (NCBI accession no.: NC_001601.1) was used. Figtree v1.4.4 was used to visualize and edit the phylogenetic tree (http://tree.bio.ed.ac.uk/software/figtree).

### Genotype Likelihood and SNP Calling

Genotype likelihoods were calculated using ANGSD v0.933 (Korneliussen et al. 2014) with the GATK model (“-GL 2”) and the following parameters: “-doMajorMinor 1 -minMapQ 20 -minQ 20 -doMaf 1 -SNP_pval 1e-3 -minMaf 0.05 -doGlf 1 -minInd 30”. Genomic regions from the reference containing repetitive elements (as described in Genome scaffolding and annotation) and having a low mappability score (*P* < 1) computed using gem (Derrien et al. 2012) were masked by setting a maximum mismatch of 4% in a 150 bp read. The same filters were used in subsequent analyses conducted with ANGSD, unless specified otherwise.

For analyses requiring higher coverage BCFtools v1.11 (Danecek et al. 2021) mpileup followed by the function was used to identify SNPs in each sample. VCFtools v.0.1.16 (Danecek et al. 2011) was used to filter the dataset to only retain biallelic SNPs with SNP quality scores (QUAL) ≥ 30 with the option “–remove-indels –max-alleles 2 –max-missing 0.9 –minQ 30 –min-meanDP 5 –max-meanDP 35 –minDP 5 –maxDP 35”. Finally, all sites included in repeated genomic regions or in regions with a low mappability score, calculated using the GEM algorithm (Derrien et al. 2012) and setting a maximum mismatch of 0.04 in a 150 bp read, were removed.

For each sample, the number of reads, percentage of aligned reads, and final mean coverage are provided in Tables S2 and S3. After filtering for mapping quality, scaffold size, sex-linked scaffolds, repetitive regions, and mappability, a total of 7,217,348 genomic sites were retained for downstream analyses, including those with low-depth data. For analyses requiring high-depth data, a subset of samples with at least 10× coverage was used, resulting in 5,850,219 sites.

### Relatedness

To detect the presence of close relatives, coefficients of relatedness were estimated with ngsRelate (Hanghøj et al. 2019) using the genotype likelihoods calculated (see above). Inferred kinship coefficients suggested that couples MED01-MED02, MED10-MED12 (MED) and BP1402-BP1602 (SVA) were siblings or parent–offspring pairs (Table S4). Thus, among these pairs, the sample with higher coverage (MED02, MED10 and BP1402) were retained for the following analyses.

### Population Structure

Genotype likelihoods from the autosomal chromosomes were used to perform several population structure analyses. Principal component analyses (PCA) were conducted in PCAngsd v0.96 (Meisner and Albrechtsen 2018) using (i) the full dataset (49 individuals), (ii) a dataset excluding SOC and NPA individuals, and (iii) the MED samples only. To estimate admixture and model-based individual clustering from genotype likelihoods, NGSadmix (Skotte et al. 2013) was ran on the complete dataset (49 individuals). The analysis was performed assuming from 1 to 10 ancestral populations (K) and doing 100 independent runs for each K. When admixture analysis converged, the maximum-likelihood result was selected. The best K was estimated using the Delta K method by Evanno et al. (2005).

Genetic clustering based on genetic distances was evaluated by building a phylogenetic tree based on an identity-by-state (IBS) matrix. The -doIBS 1 option in ANGSD (Korneliussen et al. 2014) was used to generate the IBS matrix of pairwise genetic distances, applying the same genotype likelihood settings described above. The distance matrix was then converted to the nexus format using the phangorn R package (Schliep 2011), and SPLITSTREE v4.14.6 (Huson and Bryant 2006) was used to obtain a phylogenetic network according to the Neighbor-net algorithm (Bryant and Moulton 2004).

Pairwise F_ST_ between populations was calculated by extracting F_ST_ values from the corresponding unfolded two-dimensional site frequency spectra (2DSFS), using Hudson's estimator to reduce bias when sample sizes differ among populations (Bhatia et al. 2013). Following the ADMIXTURE results, we also recalculated F_ST_ after splitting Mediterranean (MED) samples into admixed (MED_adm) and non-admixed (MED_noadm) groups. Both mitochondrial and nuclear data assigned the individual SOC02 sampled in the Sea of Cortez to the North Pacific cluster. Based on these results, we excluded this individual from the F_ST_ analysis and all subsequent analyses.

### Genomic Diversity

Genomic diversity of individuals and populations was evaluated using Watterson's theta (*θ_w_*; Watterson 1975) on callable regions. Individual-level *θ_w_* values (Fig. 3a) were estimated using Rohan (Renaud et al. 2019), which also detects runs of homozygosity (ROH). Accordingly, *θ*_W_ estimates were calculated both including ROH and excluding ROH (i.e. outside ROH) and their distribution across individuals within each population were analyzed. Population-level *θ_w_* estimates (Fig. 3b) were obtained from genotype likelihoods using ANGSD (Korneliussen et al. 2014), by estimating the site frequency spectrum (SFS) with realSFS and then computing *θ_w_* with thetaStat (sfs2theta/do_thetas). For the population-level analysis, *θ_w_* was estimated only for putatively neutral regions and for exonic regions. Neutral regions were defined as callable intergenic intervals located at least 25 kb from the closest annotated gene. Exonic regions were obtained from the reference genome annotation by merging overlapping exons across transcripts (on both strands) and intersecting them with callable regions.

### Inbreeding

Runs of homozygosity (ROH) were first identified by estimating the heterozygosity levels in 100 Kb non-overlapping windows using Rohan's probabilistic method (Renaud et al. 2019). Genomic segments were defined to be in ROH based on a Hidden Markov Model (HMM) classifier. The analysis was conducted on BAM alignments while accounting for base-calling and mapping errors. A transition/transversion rate of 2.019 was used, estimated by vcftools across the entire dataset, and an expected *θ_w_* in ROH regions (rohmu flag) of 5 × 10^−4^. Despite the method being developed to provide reliable ROH estimates for different coverages (>5×) (Renaud et al. 2019), individuals sequenced at higher coverage were downsampled to 10× in order to facilitate comparison with the other samples. ROH regions ≥ 100 Kb were used to estimate the fraction of the whole genome that was in the ROH state (FROH). The fraction of the genome in ROH obtained from the output file HMM posterior decoding using mid estimates of heterozygosity was calculated and binned by ROH size (ROH > 1 Mb).

Moreover, the sum and number of ROH (SROH and NROH, respectively) were analyzed to investigate recent demographic history, following the approach described in Foote et al. (2021). These two measures can be indicators of population size, given that they are expected to be low in large populations and when admixture is ongoing (Ceballos et al. 2018). Conversely, populations that have experienced historical bottlenecks tend to show elevated levels of both indices (Ceballos et al. 2018).

### MHC *DQB-1* Genetic Variation

To complement the genome-wide analyses with a candidate immune locus, the variation at the major histocompatibility complex (MHC) *DQB-1* gene was examined. Specifically, the exon 2 (270 bp), which is typically among the most polymorphic loci in vertebrates, was examined. High diversity at this locus is often interpreted as an indicator of adaptive potential in response to pathogen-mediated selection. MHC *DQB-1* exon 2 was analyzed for 7 MED and 2 SOC individuals from this study, as well as for 9 NPA, 6 SVA, 11 ICE, and 5 SOC individuals from previous studies. Additionally, sequences from two closely related species, *Balaenoptera musculus* (MUS, *N* = 5) and *Megaptera novaeangliae* (MEG, *N* = 5), were obtained from public repositories (Table S11). Genetic diversity indices were calculated to assess variability as previously described.

### Demographic History

Trajectories of effective population size (Ne) through time were inferred using a Multiple Sequentially Markovian Coalescent (MSMC2; Schiffels and Wang 2020) on one representative high-coverage sample for SOC, NPA, ICE and SVA populations and all samples with a coverage above 13× in MED population (Tables S2 and S3). Input data was generated using the “generate_multihetsep.py” script selecting all callable segments from 21 autosomal scaffolds. Effective population size and times were scaled using a mutation rate of 1.39 × 10−8 substitutions/site/generation and a generation time of 25.9 years (Árnason et al. 2018). To assess uncertainty, we performed 100 bootstrap replicates per sample.

### Genetic Load

Each SNP was first polarized as ancestral or derived using two outgroups: *Balaenoptera musculus* (5 individuals) and *Megaptera novaeangliae* (5 individuals) (Table S11). The ancestral allele was defined as the allele present in the two outgroups using a custom python script. All sites where at least one of these outgroups was heterozygous were discarded to maximize the confidence in the ancestral allele definition. Mutational load was assessed through two complementary approaches. All analyses were restricted to protein-coding sequences (CDS), extracted from the longest transcript of each annotated gene. Overlapping CDS regions were merged using bedtools v2.30 (Quinlan et al. 2010). First, SnpEff v5.0 (Cingolani et al. 2012) was applied to polarized SNPs located within the CDS regions to classify variants as synonymous and missense (i.e. non-synonymous). For each class, the genetic load was separated into two components: (i) the masked load assessed as the individual number of heterozygous positions, and (ii) the realized load assessed as the individual number of homozygous derived positions (Bertorelle et al. 2022). Second, relative mutational load was quantified in each individual as the number of derived alleles at sites that are under strict evolutionary constraints (i.e. highly conserved), using Genomic Evolutionary Rate Profiling scores (GERP). GERP scores for the *B. musculus* in bigwig format were obtained from a multiple alignment with 91 mammal species downloaded from ENSEMBL (https://ftp.ensembl.org/pub/release-114/compara/conservation_scores/91_mammals.gerp_conservation_score/). The *B. physalus* assembly was aligned to the *B. musculus* assembly using minimap2 with parameter -cx asm20. The GERP scores were subsequently transferred to *B. physalus* reference coordinates, using Transanno, between *B. musculus* and *B. physalus* genomes. For this analysis, heterozygous (counted as one allele) and homozygous sites (counted as two alleles) were examined taking into account that the mutational impact of heterozygous sites includes extra assumptions regarding the dominance coefficient. GERP identifies constrained elements within multiple alignments by quantifying substitution deficits, reflecting substitutions that would have occurred if the element were neutral DNA but did not due to functional constraints, accounting for phylogenetic divergence. The individual relative mutational load was calculated by summing the number of derived alleles above a GERP score of four (highly deleterious).

## Supplementary Material

evag084_Supplementary_Data

## Data Availability

The genome assembly and the Illumina reads of samples sequenced in this study were deposited in the National Center for Biotechnology Information (NCBI) with BioProject number PRJNA1268741. Genome assembly and annotation files are deposited on Zenodo (10.5281/zenodo.15649665.). Scripts are deposited on GitHub (https://github.com/rsbiello/FinWhale_popgen).
