# Supplementary material for: One Sea, Different Whales: Genomics Sheds Light on a Small Population of Fin Whales
Source: Genome Biol Evol. 2026 Apr 28;18(4):evag084. doi: 10.1093/gbe/evag084 (PMC13121172; doi:10.1093/gbe/evag084)
Supplement: evag084_Supplementary_Data [file evag084_supplementary_data.zip › FinWhale_popgen.SupFigs.11Feb26.docx]

**
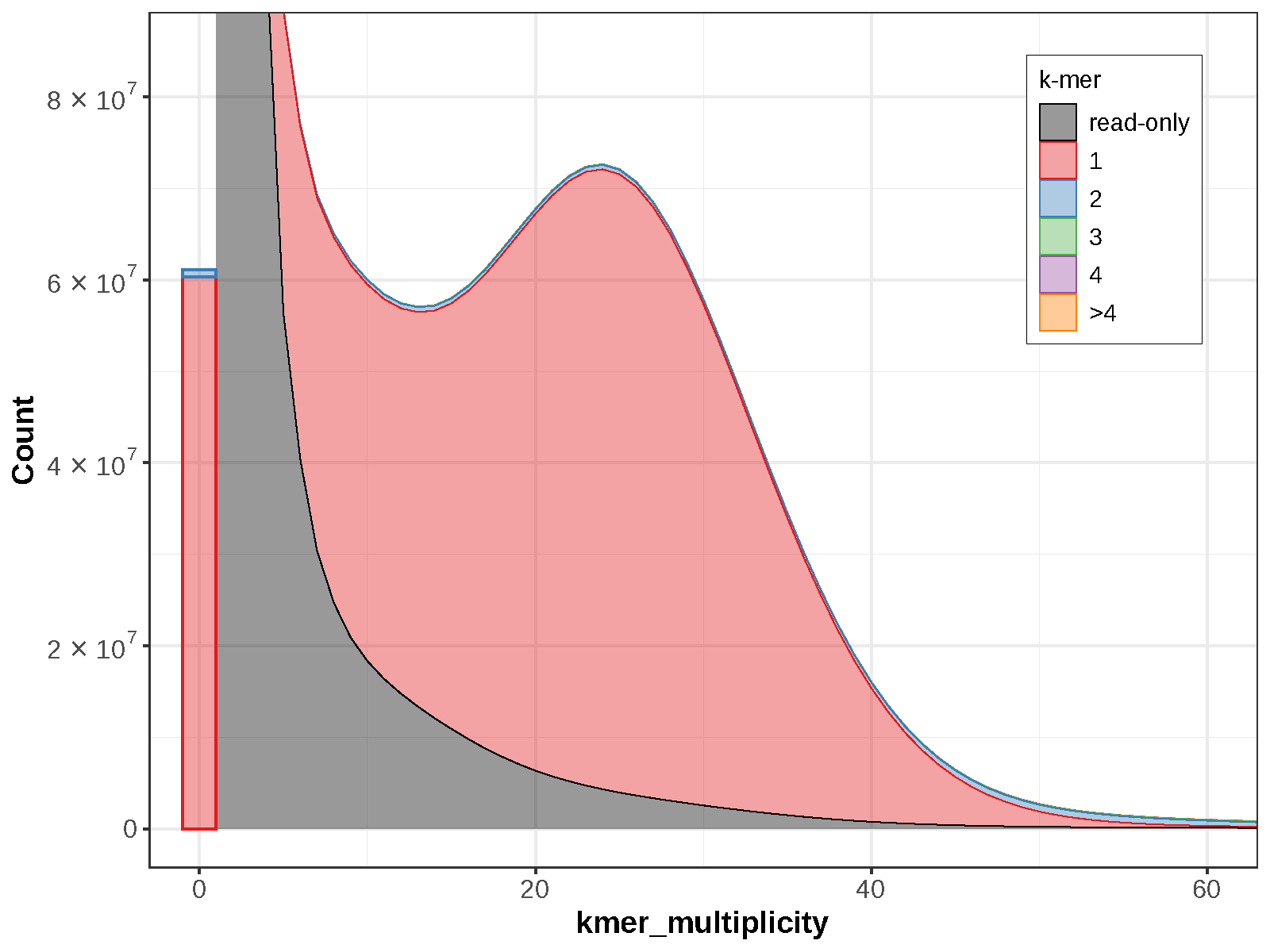
**

**Figure S1.** Merqury k-mer plots comparing k-mer content of Hi-C raw reads with Bphy_ph2.v2 assembly. The black area of the graphs represents the distribution of k-mers present in the reads but not in the assembly and the red area represents the distribution of k-mers present in the reads and once in the assembly. Other colours show k-mers found multiple times in the genome assembly.

**Figure S2.** Identification of sex chromosomes in *B. physalus*, with scaffold 6 assigned as the sex chromosome based on the mapping of genomic resequencing reads from one individual of each sex to the reference genome. The y-axis shows the ratios of male-to-female read coverages across the 22 scaffolds.


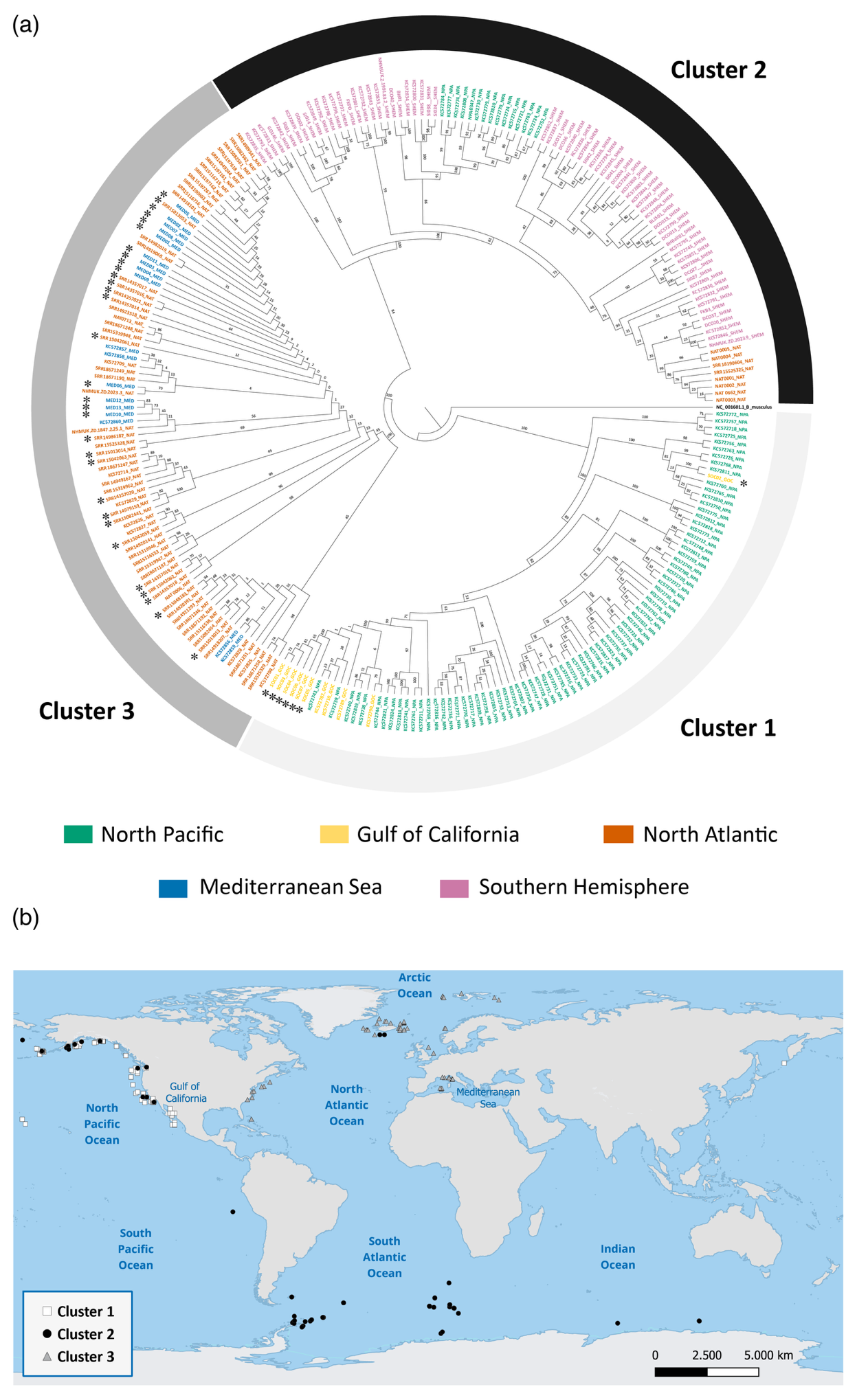


**Figure S3.**  Mitochondrial tree. a) Rooted phylogenetic tree based on the maximum likelihood of fin whale mitogenomes used in this study. Colours represent the basins of sample origin: Southern Hemisphere (pink), North Pacific Ocean (green), Gulf of California (yellow), North Atlantic Ocean (orange) and Mediterranean Sea (light blue). The numbers indicate bootstrap support values in percentage (0 to 100). The bands outside the tree were used to highlight the presence of three main clusters. The mitogenomic haplotype of *Balaenoptera musculus* (Genbank NC_001601) was used to root the tree. Individuals for which genomic data are available and used in the study are indicated by an asterisk (*). b) Geographic distribution of *Balaenoptera physalus* samples included in the mitogenomes analysis. Different shapes and colours identify different clusters in the phylogenetic tree. The map was generated using QGIS v3.28.8 (www.qgis.org).


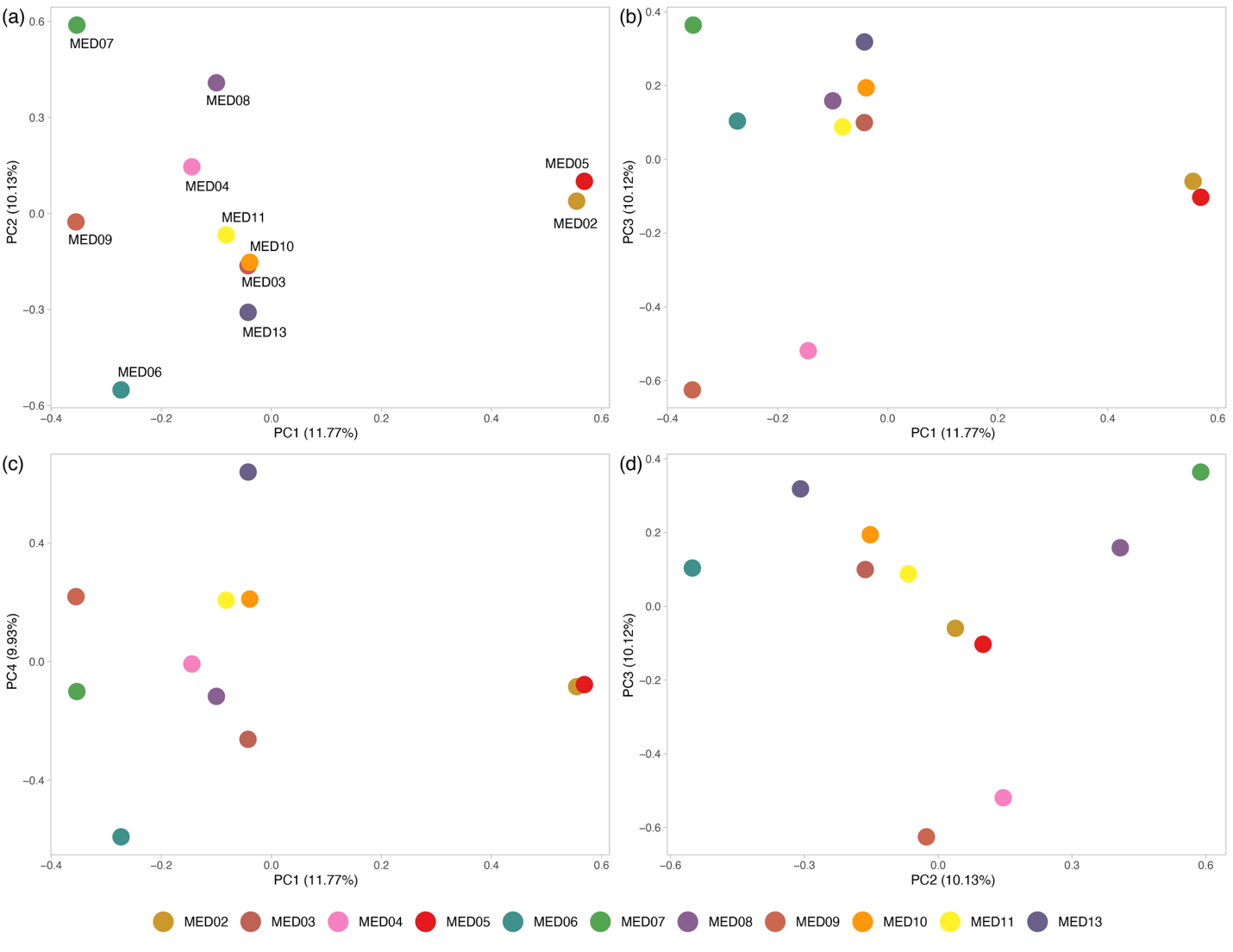


**Figure S4.** Principal component analysis (PCA) of the Mediterranean (MED) dataset showing the following component pairs: (a) PC1 vs PC2, (b) PC1 vs PC3, (c) PC1 vs PC4, and (d) PC2 vs PC3.

**Figure S5.** Splitstree network analysis of genetic relationships among 49 samples.

**Figure S6.** NgsDist distance tree inferred from autosomal genotype likelihoods for 49 samples.


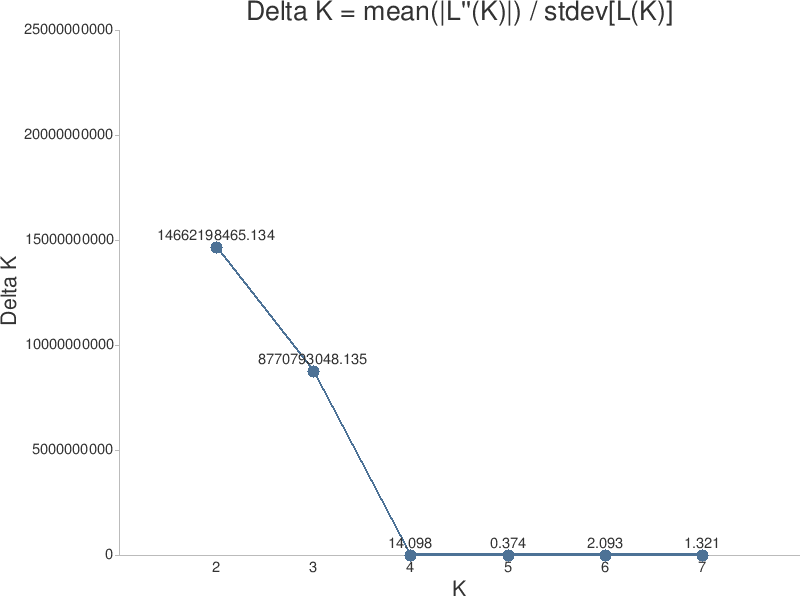


**Figure S7.** Probability of number of clusters (K) using the Delta K method by Evanno et al. (2005).


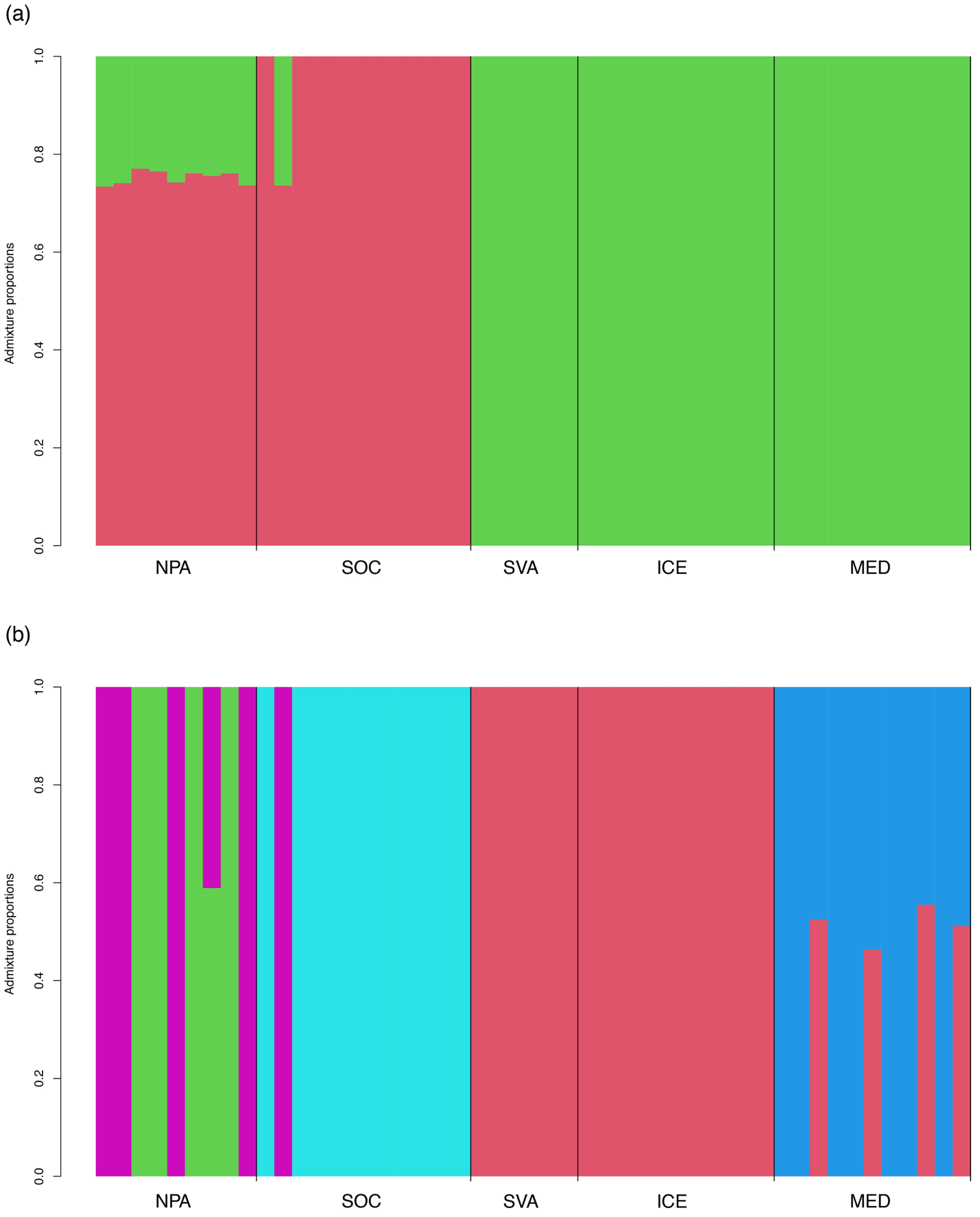


**Figure S8.** Admixture analyses. Each bar represents an individual, and each colour indicates the proportion of that individual's genome assigned to each of the K clusters. a) K2 and b) K5.

**Figure S9.** MSMC2 bootstrap results for each sample. Curves represent 100 bootstrap replicates per sample.
